# The effect of goal-directed crystalloid versus colloid administration on postoperative spirometry parameters: a substudy of a randomized controlled clinical trial

**DOI:** 10.1186/s13741-024-00381-z

**Published:** 2024-04-15

**Authors:** Mina Obradovic, Florian Luf, Christian Reiterer, Sebastian Schoppmann, Andrea Kurz, Edith Fleischmann, Barbara Kabon

**Affiliations:** 1 Department of Anaesthesia, Intensive Care Medicine and Pain Medicine, Medical University of Vienna, Spitalgasse 23, 1090 Vienna, Austria; 2 Department of Anesthesiology and Intensive Care, Hanusch Hospital, Heinrich-Collin-Straße 30, 1140 Wien, Vienna, Austria; 3 Department of Surgery, Medical University of Vienna, Spitalgasse 23, 1090 Vienna, Austria; 4 Department of Outcomes Research and General Anesthesiology, Anesthesiology Institute, Cleveland Clinic, 9500 Euclid Avenue, Cleveland, OH USA

**Keywords:** Crystalloids, Colloids, Goal-directed fluid therapy, Pulmonary function, Spirometry

## Abstract

**Background:**

Pulmonary function is impaired after major abdominal surgery and might be less impaired by restrictive fluid administration. Under the assumption of a fluid-sparing effect of colloids, we tested the hypothesis that an intraoperative colloid-based goal-directed fluid management strategy impairs postoperative pulmonary function parameters less compared to goal-directed crystalloid administration.

**Methods:**

We performed a preplanned, single-center substudy within a recently published trial evaluating the effect of goal-directed crystalloids versus colloids on a composite of major complications. Sixty patients undergoing major open abdominal surgery were randomized to Doppler-guided intraoperative fluid replacement therapy with lactated Ringer’s solution (*n* = 31) or unbalanced 6% hydroxyethyl starch 130/0.4 (*n* = 29). A blinded investigator performed bedside spirometry (Spirobank-G, Medical International Research, Rome, Italy) preoperatively as well as 6, 24, and 48 h postoperatively.

**Results:**

Median total intraoperative fluid requirements were significantly higher during crystalloid administration compared to patients receiving colloids (4567 ml *vs.* 3044 ml, *p* = 0.01). Six hours after surgery, pulmonary function parameters did not differ significantly between the crystalloid — and the colloid group: forced vital capacity (FVC): 1.6 l (1.2–2 l) *vs*. 1.9 l (1.5–2.4 l), *p* = 0.15; forced expiratory volume in 1 second (FEV1): 1.1 l (0.9–1.6 l) *vs.* 1.4 l (1.2–1.7 l), *p* = 0.18; and peak expiratory flow (PEF): 2 l.sec^−1^ (1.5 – 3.6 l.sec ^−1^) vs. 2.3 l.sec ^−1^ (1.8 – 3.4 l.sec ^−1^), *p* = 0.23. Moreover, postoperative longitudinal time × group interactions of FVC, FEV1, and PEF between 6 and 48 postoperative hours did not differ significantly.

**Conclusion:**

Postoperative pulmonary function parameters were similarly impaired in patients receiving goal-directed crystalloid administration as compared to goal-directed colloid administration during open abdominal surgery.

**Trial registration:**

ClinicalTrials.gov (NCT00517127, registered on August 16, 2007) and EudraCT (2005-004602-86).

## Background

Pulmonary function is significantly impaired after major abdominal surgery Treschan et al. (2017); Treschan et al. (2012). Consequently, the risk of complications such as pneumonia, respiratory failure, and prolonged hospitalization increases (Treschan et al. 2017; Qaseem et al. 2006; Brooks-Brunn 1995). Formation of atelectasis (Grigor 1954; Hedenstierna and Edmark 2005), as well as fluid overload with degradation of the endothelial surface layer and subsequent tissue edema, might contribute to postoperative pulmonary dysfunction (Brandstrup et al. 2003; Holte et al. 2002). A restricted fluid regimen improved postoperative pulmonary function compared to liberal fluid administration after colonic surgery (Holte et al. 2007). Even in healthy volunteers, not exposed to surgical stress and without altered capillary permeability, a liberal infusion rate caused deterioration of pulmonary function (Holte et al. 2003).

Perioperative fluid management represents a major determinant of postoperative morbidity (Brandstrup et al. 2003; Thacker et al. 2016; Nisanevich et al. 2005). Intraoperative individualized goal-directed fluid administration based on advanced hemodynamic monitoring aims to optimize cardiac performance and oxygen delivery while preventing iatrogenic hyperhydration and its harmful consequences (Makaryus et al. 2018). Some evidence suggests that goal-directed fluid therapy might beneficially effect short- and long-term mortality, overall complication rate, and recovery of gastrointestinal function when compared to conventional fluid therapy in high risk (Sun et al. 2017) as well as low to medium risk surgical patients (Calvo-Vecino et al. 2018). Nevertheless, available data are inconsistent (Sun et al. 2017; Pearse et al. 2014). Specifically, the most effective treatment strategy regarding various patient populations and interventions, monitoring tools, target parameters, algorithms, and types of fluid remains controversial (Wilms et al. 2014).

In most goal-directed studies, a colloid-based algorithm has been used for fluid optimization. Colloids offer favorable properties in regard to plasma expansion and intravascular retention time (Niemi et al. 2010). Intraoperative goal-directed use of a balanced hydroxyethyl starch solution was associated with better hemodynamic stability and significantly less infusion requirements compared to crystalloids only (Feldheiser et al. 2013; Yates et al. 2014). However, a recently published randomized controlled trial did not demonstrate any beneficial effect of goal-directed colloid administration compared to crystalloid administration on a composite of postoperative major complications and duration of hospitalization (Kabon et al. 2019). Within the present preplanned substudy of the abovementioned trial, we tested the hypothesis that an intraoperative colloid-based goal-directed fluid regimen preserves postoperative pulmonary function parameters better as compared to goal-directed crystalloid administration.

## Methods

This prospective, parallel group, randomized, controlled, double-blinded trial was performed at the Department of Anaesthesia, Intensive Care Medicine and Pain Medicine of the Medical University of Vienna. The study was approved as a part of a large multicenter outcome trial evaluating the effect of goal-directed administration of crystalloids or colloids on a composite of postoperative complications and morbidity (Kabon et al. 2019). The trial was approved by the local ethics committee (EK 431/2005), registered at ClinicalTrials.gov (NCT00517127) and EudraCT (2005-004602-86), and conducted in accordance with the Declaration of Helsinki and good clinical practice. Written informed consent was obtained from all participants. This manuscript adheres to the applicable CONSORT guidelines.

We included patients scheduled for elective moderate- to high-risk open abdominal surgery with an expected duration of at least 2 h who were aged between 18 and 80 years, were American Society of Anesthesiologists physical status I-III, and had a body mass index (BMI) of less than 35 kg.m^−2^. We excluded patients who had compromised kidney function (estimated creatinine clearance less than 30 ml.min^−1^), estimated cardiac ejection fraction less than 35%, severe chronic obstructive pulmonary disease, coagulopathies, or known esophageal or aortic abnormalities.

### Anesthetic management

General anesthesia was induced with 1–3 μg.kg^−1^ fentanyl, 2–3 mg.kg^−1^ propofol, and 0.6 mg.kg^−1^ rocuronium. Following intubation, anesthesia was maintained with sevoflurane (up to 1.5 mean alveolar concentration) in a carrier gas of 80% inspired oxygen. According to patients’ requirements, additional fentanyl and non-depolarizing neuromuscular blocking agents were administered throughout surgery. Standard monitoring included electrocardiography, noninvasive arterial blood pressure measurement, pulse oximetry, and esophageal core temperature monitoring. After induction of anesthesia, all patients received an arterial line; central venous catheters were placed as deemed clinically necessary. We performed pressure-controlled mechanical ventilation with tidal volumes between 6 and 8 ml.kg^−1^ ideal body weight and a positive end-expiratory pressure (PEEP) of 5 mmHg. Ventilatory rate was adjusted to keep end-tidal carbon dioxide levels of 35–40 mmHg, and inspiratory to expiratory time ratio was set at 1:1.7. Maintained a hematocrit level > 30% in patients with known cardiovascular disease and age > 65 years, 28% in patients with one or the other, and 26% in the others. We actively warmed all patients with convective warming to maintain perioperative normothermia. At the end of surgery and after complete reversal of the neuromuscular blockade, patients were extubated following manual hyperinflation with a maximal pressure of 30 mmHg, while no further recruitment maneuvers were performed during surgery. Patients were transferred to postoperative care unit (PACU) or intensive care unit (ICU) at the discretion of the attending anesthesiologist.

### Randomization and fluid management

Shortly before induction of anesthesia, patients were randomized 1:1 to either additional goal-directed bolus administration of crystalloids (lactated Ringer’s solution, Fresenius Kabi, Germany) or goal-directed bolus administration of colloids (hydroxyethyl starch 6% 130/0.4, Voluven, Fresenius-Kabi, Germany). The randomization sequence was generated by the study statistician using the PLAN procedure in SAS statistical software (SAS Institute, USA) using randomly sized blocks. A trained study coordinator evaluated eligibility, obtained informed consent, and then on the day of surgery before induction of anesthesia enrolled the participants by opening the concealed envelope. Intraoperative investigators and clinicians were not blinded to treatment. However, an observer strictly blinded to group assignment performed postoperative spirometry.

All patients were given 5–7 ml.kg^-1^ of lactated Ringer’s solution during induction of anesthesia followed by 3–5 ml.kg^−1^.h^−1^ for maintenance, normalized to ideal body weight, throughout surgery. We calculated the ideal body weight according to the Robinson formula (Robinson et al. 1983). Thereafter, the randomized fluid, crystalloid, or colloid was administered esophageal Doppler (Cardiac Q, Deltex Medical Group PLC, Chichester, UK) guided according to a previously published algorithm (Gan et al. 2002). A 250-ml aliquot of lactated Ringer’s solution or 6% hydroxyethyl starch was administered when corrected flow time (FTc) was less than 0.35 s. If stroke volume (SV) increased ≥ 10% and FTc still remained below 0.35 s, the bolus was repeated until no further increase in stroke volume was observed. If FTc increased above 0.35 s, no further fluid challenge was administered, and measurements were repeated after 10 min. If FTc remained low after bolus administration and SV did not increase by ≥ 10%, no further bolus was administered, and measurements were repeated after 10 min. When we observed a further decrease in SV by at least 10% of the last measured value, the fluid challenge was repeated. In case of a mean arterial blood pressure (MAP) below 65 mmHg and no Doppler-detected signs of hypovolemia, intravenous vasopressors were administered at the discretion of the attending anesthesiologist.

### Postoperative care and spirometry

All patients received postoperative care according to clinical standard. Patients received 2 ml. kg^−1^.h^−1^ crystalloids with additional fluid as deemed clinically necessary for 2 h. Subsequently, fluid management was performed at the discretion of the attending physicians. We administered supplemental oxygen via a Venturi mask to maintain oxygen saturation above 96%. All study participants received intravenous patient-controlled analgesia (PCA). Patients were able to administer a bolus of 2.5-mg morphine when needed. No basal rate was set, and the lock out time was 10 min with a maximal dosage of four boluses per hour. Postoperative pain evaluation was performed with visual analogue scale, ranging from 0 (no pain) to 10 (worst pain imaginable). Scores were evaluated at rest and at effort, while patients were performing pulmonary function tests.

Pulmonary function was evaluated with a bedside spirometer (Spirobank-G™ Medical International Research, Rome, Italy) by a blinded investigator. Preoperatively, the requested tasks were demonstrated for patients in order to comprehend the correct technique. Then a clean, disposable mouthpiece was attached to the spirometer and a nose clip to the patients’ nose. Under detailed instructions, patients performed the tests to obtain values of forced vital capacity (FVC), forced expiratory volume in 1 second (FEV1), and peak expiratory flow (PEF). During performance of spirometry tests, patients were encouraged to inhale completely and exhale maximally until no more air could be breathed out. All tests were performed in a sitting position. Measurements were performed the day before surgery as baseline (T_0_) as well as after 6, 24, and 48 postoperative hours (T_1_–T_3_).

### Measurements and outcomes

Patients’ demographic and morphometric data were recorded. We recorded all routine anesthetic, respiratory, and hemodynamic variables at 10-min intervals. Detailed records of intraoperative fluid balances including urinary output and estimated blood loss were kept. Arterial blood gases were obtained at least hourly during surgery, at arrival at the PACU or ICU, and during the first six postoperative hours according to clinical requirements. Postoperative fluid balances were recorded from time of arrival at the PACU or ICU until the second postoperative day.

Primary outcomes were FVC, FEV_1_, and PEF evaluated with means of bedside spirometry 6 h after extubation (T_1_). Secondary outcomes were summary measures of FVC, FEV_1_, and PEF until 48 postoperative hours (T_1_–T_3_). We also recorded supplemental oxygen requirements, postoperative pain scores, and morphine requirements at T_1_–T_3_.

### Sample size calculation and statistical analysis

When the trial was initiated, no specific data about the fluid-sparing effect of goal-directed administered colloids versus crystalloids were available. Sample size calculation was thus based on results of a trial by Holte et al., which compared the effect of a restrictive and a liberal fluid regimen on postoperative pulmonary function and which showed a difference in FVC of approximately 0.5 l with standard deviations near 0.5 l 6 h postoperatively (Holte et al. 2007a). In this study, the ratio between the amounts of administered fluid in the two groups was approximately 1:3. This corresponded with our expected difference between crystalloids and colloids based on traditional doctrine. Assuming similar effects for goal-directed colloid administration, a sample size of 22 patients in each group was calculated, allowing a type I error of 5% and a type II error of 10%. To compensate for potential dropouts, we thus included 60 patients.

Groups were compared for balance in patients’ demographic data, intraoperative characteristics, and postoperative variables. Absolute standardized differences (ASD) were calculated for baseline characteristics and baseline spirometry data. Any variable with an absolute standardized difference > 0.20 (defined as small effect size) was considered imbalanced and thus included as a covariate in post hoc multivariable linear regression models with the primary outcome parameters as the dependent variable. Serial measurements of intraoperative parameters were averaged for each patient separately and then averaged among the patients in each treatment groups. Normal distribution was assessed with q-q plot and Kolmogorov-Smirnov test. Continuous data were compared using unpaired two-tailed *t*-tests, when values were normally distributed. Wilcoxon rank-sum test was used for continuous data, which were not normally distributed. Nominal data were analyzed with either chi-square or Fisher’s exact test. Paired comparisons between baseline data and primary outcome data were performed with Wilcoxon signed-rank test. Data were presented as means ± SD, medians (IQR), or as numbers (proportions) as appropriate.

We compared spirometry parameters 6 h after surgery and evaluated the differences in time courses of FEV1, FVC, and PEF between the two randomized groups using nonparametric analysis for longitudinal data in factorial designs as proposed by Brunner et al. Regarding our three different primary endpoints (FVC, FEV1, and PEF), a Bonferroni-corrected *p*-value < 0.016 was considered to be statistically significant. For all other variables, a *p*-value < 0.05 was considered to be statistically significant.

Analyses were conducted with SPSS software (IBM SPSS Statistics for Macintosh, Version 24.0. Armonk, NY, USA); R for Macintosh, Version 3.2.1 (https://www.R-project.org/) was used to calculate ASD.

For evaluating the time × group interaction as well as the within-group trend, the R-package nparLD, release 4.2.2 was used.

## Results

**Fig. 1:**
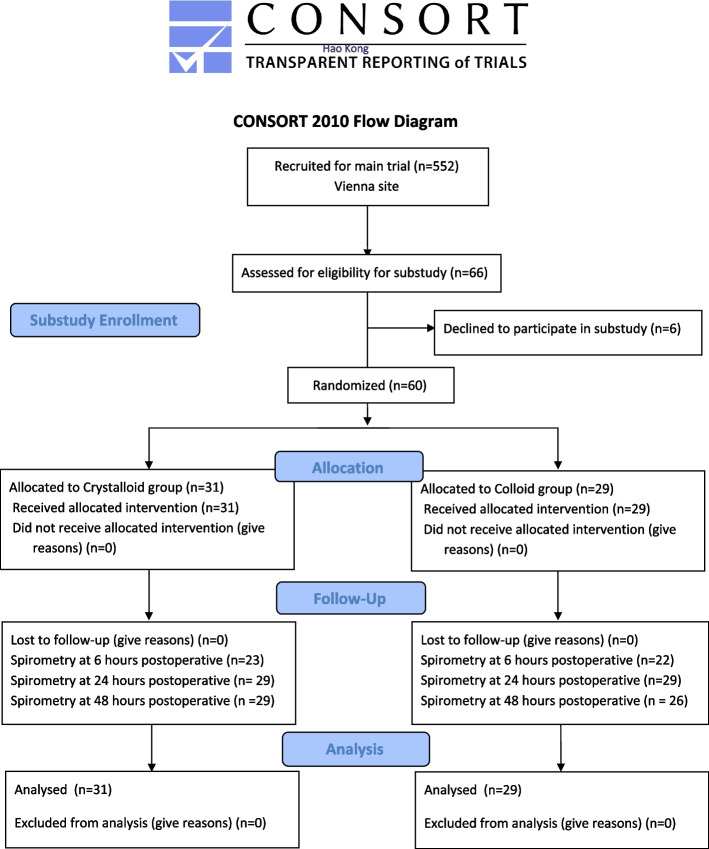
Study flow chart

Between April 2008 and November 2009, we included 60 patients (31 were randomized to the crystalloid group, while 29 received colloids) (Fig. 1). In 45 patients (23/22), spirometry was performed at T_1_. At T_2_ and T_3_, 58 (29/29) and 55 (29/26) measurements were completed, respectively. Reduced consciousness and lack of willingness were the main reasons for not fulfilling the tasks.

Other than ASA scores, which were higher in the colloid group, and duration of surgery, which was approximately 40 min shorter in the colloid group, baseline characteristics were well balanced between the groups (Table 1).

**Table 1:** Baseline and intraoperative characteristics

Factors and variables	Crystalloid *(n = 31)*	Colloid *(n = 29)*	*p-value*	*ASD*
Age; *yrs*	52 ± 13	53 ± 17		0.06
Height; *cm*	172 ± 8	171 ± 9		0.01 1
Weight; *kg*	75 ± 11	77 ± 21		0.15
BMI; *kg/m*^*2*^	25 ± 4	26 ± 5 16/13		0.11
Sex (m/f); *n*	18/13			0.06
ASA score (1/2/3); *n*	11/17/3	7/16/6		0.34
Smoking history (yes/no); *n*	8/23	6/23		0.12
Type of surgery (upper abdominal/colorectal); *n*	21/10	21/8		0.10
Liver resection; *n*	9	18		
Pancreaticoduodenectomy; *n*	8	1		
Rectal surgery; *n*	6	6		
Colectomy; *n*	3	1		
Left pancreatectomy; *n*	2	2		
Sigma resection; *n*	1	1		
Total pancreatectomy; *n*	1	0		
Sarcoma resection; *n*	1	0		
Duration of surgery; *min*	267 ± 106	228 ± 92		0.39
Intraoperative variables				
TWA MAP; *mmHg*	71 ± 8	78 ± 9	0.51a	
TWA HR; *bpm*	70 ± 12	72 ± 12	0.78a	
TWA CVP; *mmHg*	8 ± 2	10 ± 3	0.14a	
TWA FTc; *msec*	353 ± 22	359 ± 21	0.45a	
TWA SV; *ml*	78 ± 17	80 ± 16	0.99a	
TWA end-tidal sevoflurane; *%*	2 (1.8–2.1)	1.8 (1.7–2)	0.02b	
Fentanyl, μg	1006 ± 423	847 ± 353	0.63a	
Total fluid intake; *ml*^c^	4567 (3500–5800)	3044 (2125–3825)	0.01b	
Crystalloids; ml	4100 (3025–5041)	1300 (955–2000)	< 0.001b	
Colloids; *ml*	N.a.	1250 (750–1500)	N.a.	
Number of boluses; *n*	9 (6–12)	5 (3–6)	< 0.001b	
Vasopressor therapy (yes/no); *n*	23/8	13/16	0.03d	
Phenylephrine; *mg*	0.32 (0.14–0.8)	0.29 (0.18–0.56)	1.00b	
Norepinephrine; *mg*	0.1 (0.06–0.12)	0.02	1.00b	
Urinary output; *ml*	300 (200–450)	300 (135–475)	0.83b	
Blood loss; *ml*	400 (175–600)	300 (150–1000)	0.59b	
TWA BE; *mmol/l*	−1.9 ± 1.9	−2.1 ± 2	0.63a	
TWA FiO_2_, %	79 (78–80)	79 (77–80)	0.94b	
PaO_2_ (start of surgery); *mmHg*	366 (324–453)	400 (305–458)	0.30b	
PaO_2_/FiO_2_ ratio; *mmHg*	454 (385–480)	453 (383–516)	0.70b	
PaO_2_ (end of surgery); mmHg	381 (326–429)	392 (359–428)	0.80b	
PaO_2_/FiO_2_ ratio; *mmHg*	462 (404–500)	488 (443–526)	0.37b	
PaCO_2_; *mmHg*	40 ± 3	41 ± 3	0.55a	

Data are presented as mean ± SD, median (IQR), or number. Abbreviations: *ASA* American Society of Anesthesiologists, *ASD* absolute standardized differences, *BMI* body mass index, *CVP* central venous pressure, *f* female, *FiO*_*2*_ fraction of inspired oxygen concentration. *FTc* corrected flow time, *HR* heart rate, *m* male, *MAP* mean arterial pressure, *PaCO*_*2*_ arterial partial pressure of carbon dioxide, *PaO*_*2*_ arterial partial pressure of oxygen, *SV* stroke volume, *TWA* time-weighted average. ^a^Unpaired, two-tailed *t*-test. ^b^Wilcoxon rank-sum test. ^c^Total fluid intake includes baseline, fluid boli, antibiotics, analgesics, and additional fluid, administered at the discretion of attending anesthesiologist. ^d^Fisher’s exact test

Hemodynamic as well as Doppler-derived parameters did not differ. Total intraoperative fluid input including additional fluid, antibiotics, and analgesics was significantly higher in the crystalloid group compared to the colloid group: 4567 ml (3500–5800 ml) *vs* 3044 ml (2125–3825 ml), *p* = 0.01. Patients assigned to the crystalloid group received 4100 ml (3025–5041 ml) of lactated Ringer’s solution, while patients in the colloid group received 1300 ml (955–2000 ml) of lactated Ringer’s solution and 1250 ml (750–1500 ml) of hydroxyethyl starch solution. Specifically, patients in the crystalloid group obtained 9 (6–12) boluses of the study fluid, while patients in the colloid group obtained 5 (3–6) boluses. Significantly, more patients in the crystalloid group required intravenous vasopressor support compared to the colloid group. Urinary output and blood loss were comparable; no blood transfusions were required (Table 1).

**Fig. 2:**
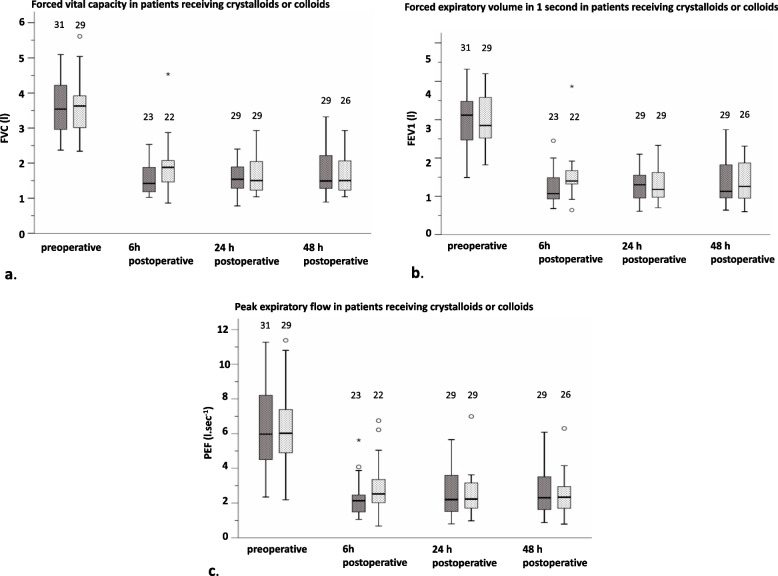
**a** Boxplot of forced vital capacity (FVC) for group and time in patients receiving crystalloids (dark boxes) or colloids (light boxes). °Represents outliners, *represents extreme outliners, numbers represent number of patients performing spirometry. No significant difference in the time trend from 6 to 48 h was found between the groups (*p* = 0.38). Furthermore, the difference in time trend from 6 to 24 h (*p* = 0.2) did not show a significant result. **b** Boxplot of forced expiratory volume in 1 second (FEV1) for group and time in patients receiving crystalloids (dark boxes) or colloids (light boxes). °Represents outliners, *represents extreme outliners, numbers represent number of patients performing spirometry. No significant difference in the time trend from 6 to 48 h was found between the groups (*p* = 0.32). Furthermore, the difference in time trend from 6 to 24 h (*p* = 0.2) did not show a significant result. **c** Boxplot of peak expiratory flow (PEF) for group and time in patients receiving crystalloids (dark boxes) or colloids (light boxes). °Represents outliners, *represents extreme outliners, numbers represent number of patients performing spirometry. No significant difference in the time trend from 6 to 48 h was found between the groups (*p* = 0.12). Furthermore, the difference in time trend from 6 to 24 h (*p* = 0.193) did not show a significant result

While intraoperative opioid requirements did not differ, end-tidal sevoflurane concentrations were statistically significantly higher in the crystalloid group. However, this small absolute difference of 0.2 vol% is most likely of no clinically relevance. Fraction of inspired oxygen concentration (FiO_2_) was well controlled and did not differ between the groups. Arterial partial pressures of oxygen (PaO_2_) and Horowitz quotients (PaO_2_/FiO_2_ ratio) at the start of surgery as well as intraoperative carbon dioxide partial pressures (PaCO_2_) were comparable in both groups (Table 1). Also, our different fluid management strategies did not affect PaO_2_ and PaO_2_/FiO_2_ ratio at the end of surgery. Postoperative fluid balances did not differ between the groups. Visual analogue scores for pain were comparable between the groups at all postoperative time points of evaluation, at rest as well as with effort. Also, morphine requirements did not differ at any given point in time. Six hours after surgery, more patients in the crystalloid group compared to the colloid group required oxygen insufflation, whereas arterial oxygen levels were comparable: 115 mmHg (87–136 mmHg) *versus* 104 mmHg (99–142 mmHg), *p* = 0.94. Also, on the first postoperative day, more patients in the crystalloid group received oxygen therapy. However, differences on both days were not statistically significant. On the second postoperative day, an equal number of patients in each group required oxygen insufflation (Table 2). In a post hoc analysis, postoperative PaO_2_ values in patients, who did not fulfill the tasks 6 h after surgery, did not differ significantly compared to patients who were able to perform spirometry: 121 (92–144) mmHg versus 110 (100–134) mmHg, *P* = 0.62. Measurements of FVC, FEV1, and PEF did not differ significantly preoperatively as well as 6 h after surgery between the groups. All parameters significantly declined 6 h after surgery compared to baseline measurements within both groups (Table 3). Moreover, postoperative longitudinal time × group interactions of FVC, FEV1, and PEF between 6 and 48 postoperative hours did not differ significantly (Fig. 2a–c). When we adjusted for both imbalanced covariates, ASA scores and duration of surgery, differences in our primary outcome parameters FVC, FEV1, and PEF were found again non-significant (*p* = 0.37, 0.65, and 0.93, respectively).

**Table 2:** Postoperative variables

Postoperative variables	Crystalloid *(n = 31)*	Colloid *(n =29)*	*p*-value
*Postoperative fluid balances*			
Postoperative intake; *ml*^*a*^	2800 (1440–3800)	3001(2558–3865)	0.17b
Postoperative output; *ml*^*a*^	1560 (920–1920)	1410 (1120–1920)	0.86b
1st postoperative day intake; *ml*	3450 (2760–4225)	3375 (2988–3870)	0.90b
1st postoperative day output; *ml*	2670 (1768–3118)	2275 (1720–3072)	0.62b
2nd postoperative day intake; *ml*	3325 (2595–3850)	3340 (2990–3840)	0.67b
2nd postoperative day output; *ml*	2245 (1610–3612)	2500 (1950–3370)	0.55b
*6 h postoperative*			
Oxygen requirements (yes/no); *n*	23/8	15/14	0.11c
VAS for pain during rest	4 (3–5)	3 (3–4)	0.22b
VAS for pain during effort	5 (4–7)	5 (4–8)	0.87b
Cumulative PCA requirements; *mg*^*d*^	15 (7–24)	19 (9–25)	0.55b
*24 h postoperative*			
Oxygen requirements (yes/no); *n*	12/19	8/21	0.42c
VAS for pain during rest	3 (1–5)	2 (1–3)	0.13b
VAS for pain during effort	5 (3–7)	4 (3–7)	0.22b
Cumulative PCA requirements; *mg*^*d*^	38 (20–60)	43 (26–59)	0.58b
*48 h postoperative*			
Oxygen requirements (yes/no); *n*	2/29	2/27	1.00c
VAS for pain during rest	3 (2–4)	2 (0–3)	0.25b
VAS for pain during effort	4 (3–5)	3 (3–6)	0.86b
Cumulative PCA requirements; *mg*^*d*^	55 (30–73)	48 (36–86)	0.89b

Data are presented as median (IQR) or number. Abbreviations: *PCA* patient-controlled analgesia, *VAS* visual analogue scale. ^a^Postoperative intake and output represent oral and intravenous fluid intake and urinary output from immediately after surgery until 6 a.m. on the first postoperative morning. ^b^Wilcoxon rank-sum test. ^c^Fisher’s exact test. ^d^Cumulative morphine requirements are presented 6, 24, and 48 h postoperatively

**Table 3:** Spirometry data

Baseline and outcome data	Crystalloid *n = 31*	Colloid *n = 29*	*p-value*	ASD
Preoperative FVC; *l*	3.7 (3–4.5)	3.6 (3–4)	0.56a	0.14
Preoperative FEV1; *l*	3.1 (2.4–3.6)	2.9 (2.5–3.6)	0.50a	0.19
Preoperative PEF; *l.sec*^*−1*^	6.4 (4.5–8.3)	6 (4.7–7.5)	0.73a	0.11
	***n = 23***	***n = 22***		
6-h postoperative FVC; *l*	1.6 (1.2–2)b	1.9 (1.5–2.4)b	0.15a	
6-h postoperative FEV1; *l*	1.1 (0.9–1.6)b	1.4 (1.2–1.7)b	0.18a	
6-h postoperative PEF; *l.sec*^*−1*^	2 (1.5–3.6)b	2.3 (1.8–3.4)b	0.23a	
	***n = 29***	***n = 29***		
24-h postoperative FVC; *l*	1.5 (1.3–2)	1.5 (1.2–2.1)	0.82a	
24-h postoperative FEV1; *l*	1.3 (0.9–1.7)	1.3 (1–1.7)	0.92a	
24-h postoperative PEF; *l.sec*^*−1*^	2.2 (1.6–3.7)	2.3 (1.8–3.4)	0.64a	
	***n = 29***	***n = 26***		
48-h postoperative FVC; *l*	1.5 (1.3–1.9)	1.8 (1.4–2.3)	0.97a	
48-h postoperative FEV1; *l*	1.2 (0.9–1.9)	1.3 (1–1.9)	0.41a	
48-h postoperative PEF; *l.sec*^*−1*^	2.7 (1.6–3.9)	2.4 (1.7–3)	0.39a	

Data are presented as median (IQR). Abbreviations: *ASD*, absolute standardized differences; *FEV1*, forced expiratory pressure in 1 second; *FVC*, forced vital capacity; *PEF*, peak expiratory flow. ^a^Wilcoxon rank-sum test. ^b^Wilcoxon signed-rank test, significantly different from preoperative baseline, *p* < 0.001

## Discussion

Goal-directed colloid administration did not lead to significantly less impaired postoperative pulmonary function compared to goal-directed crystalloid administration as evaluated by bedside spirometry in patients undergoing major open abdominal surgery. This is consistent with a prospective randomized trial in patients undergoing laparoscopic gynecologic surgery. In this study, the administration of equal and fixed rates of crystalloids or colloids did not show any differences in regard to postoperative pulmonary function parameters (Hayes et al. 2012). In contrast, we compared two physiologically similar, equieffective fluid-management strategies in regard to postoperative pulmonary function. Patients randomized to colloids required significantly less total intraoperative fluid to reach similar hemodynamic endpoints compared to patients receiving only crystalloids. We confirmed the previously observed fluid-sparing effect of colloid administration within a goal-directed setting (Feldheiser et al. 2013; Yates et al. 2014; Joosten et al. 2018). Noteworthy, the abovementioned studies used different monitoring systems, hemodynamic algorithms, or rescue fluids. Thus, direct comparisons between studies might be restrained. However, in our main trial, the difference between the amount of fluid given in both study groups was considerably smaller compared to the present substudy. Compared to our main trial, duration of surgery was approximately 1 h longer, and estimated blood loss was slightly higher in this specific subgroup. This could be a possible explanation for the increased fluid requirements especially in the crystalloid group. Due to prolonged surgical trauma and stress response, degradation of endothelial vascular barrier and thus increased fluid shifts might have occurred (Chappell et al. 2008). This is also reflected by the fact that significantly more patients in the crystalloid group required vasopressor support. In turn, one might hypothesize that a more frequent vasopressor use might have led to a higher arterial blood pressure and thus to a reduced amount of fluid administration in the crystalloid group. However, we primarily used flow-related parameters to guide our fluid management. Thus, undeliberate vasopressor administration might have led to a shorter aortic flow time due to vasoconstriction and thus in turn most likely to further fluid challenge tests. As we described in the protocol, we administered vasopressor therapy only whenever blood pressure was below our treatment threshold after fluid volume optimization. As this algorithm was strictly followed in both groups, we do not assume any impact on our outcome.

Remarkably, median total fluid administration in our crystalloid group (4567 ml) resembled the amounts of overall intraoperative fluid intake in the liberal regimen (4400 ml) in a study of Holte and colleagues (Holte et al. 2007b). In this study, a liberal fluid regimen was associated with a significant impairment of pulmonary function compared to fluid restriction after colonic resection (Holte et al. 2007a).

Nevertheless, despite a difference of roughly 1.5 l of total fluid intake between our two groups, we could not demonstrate a comparable effect on postoperative pulmonary function.

A possible explanation might be the timing of fluid administration. As long as fluid is administered at the right time in order to achieve a specific hemodynamic endpoint, the cumulative amount or type of fluid might not matter. During our Doppler-guided management, additional fluid boluses were given according to individual requirements, whenever hypovolemia was detected, while Holte used fixed rates. One might hypothesize that liberal fluid administration without guidance according to individual requirements might more likely lead to overhydration and interstitial edema due to destruction of the endothelial surface layer (Chappell et al. 2008). Accordingly, pulmonary and bronchial congestion might occur, contributing to postoperative restrictive lung impairment and small airway obstruction (Pellegrino et al. 1985). On the other hand, individualized goal-directed fluid therapy might prevent hyperhydration and consequently might inhibit degradation of the endothelial glycocalyx and further deterioration of the vascular barrier and thus maintain adequate pulmonary function.

However, there are some controversial studies, showing transient improvements in pulmonary function after liberal fluid regimens in patients undergoing knee arthroplasty and laparoscopic cholecystectomy (Holte et al. 2007b; Holte et al. 2004). A reasonable explanation for this discrepancy might be a different level of surgical stress. In minor surgical procedures, stress response is negligible compared to major open abdominal surgery. Larger procedures are associated with more profound changes in fluid hemostasis, which causes greater fluid shifts and thus interstitial fluid conservation (Holte et al. 2002). Consistent with this theory, pulmonary function is less compromised after laparoscopic *versus* open surgery (Putensen-Himmer et al. 1992).

In general, pulmonary function is impaired after mechanical ventilation during general anesthesia (Tiefenthaler et al. 2011). Unsurprisingly, postoperative spirometry parameters worsened significantly in both groups after six postoperative hours and stayed diminished during the entire investigation period. Our observed absolute values as well as the decline of postoperative parameters close to 50% from baseline correspond with previously published spirometry results obtained in similar patient populations undergoing open abdominal procedures (Treschan et al. 2017; Treschan et al. 2012).

Our sample size was calculated to show a difference of 0.5 l in FVC 6 h postoperatively, and did not reveal any significant findings regarding a benefit of colloid administration, as primarily expected. Nevertheless, we cannot rule out that a lower total fluid intake might have contributed to an initial lesser impairment of lung function parameters in the colloid group of roughly 300 ml at T_1_. However, our study was not powered to detect such minor differences, which might not be of clinical relevance.

There was a tendency of increased postoperative oxygen requirements up to 24 h postoperatively in the crystalloid group, which was not statistically significant. Considering that our patient population was fairly young and healthy without any preexisting pulmonary disease, this finding might be more relevant or significant in high-risk patient populations, such as obese patients, elderly patients, or patients with chronic pulmonary disease. This is also reflected by a very low overall pulmonary complication rate in our main trial with 3% in the colloid groups versus 5% in the crystalloid group (Kabon et al. 2019).

Spirometry parameters only represent surrogates of pulmonary morbidity (Ballantyne et al. 1998). Moreover, our postoperative spirometry results might have been affected by other factors besides overhydration such as pain, fatigue, enhanced mobilization, or formation of atelectasis. Furthermore, we found no evidence that a lower PaO_2_, potentially due to fluid overload, lung edema, and thus impaired alveolar-arterial diffusion, was a cause for not participating on spirometry measurements 6 h after surgery. We did not prospectively record mobilization time; however, no early mobilization programs have been established, and mobilization was performed according to institutional standard in all patients on the first postoperative day. Thus, mobilization efforts did not affect our measurements performed 6 h after surgery. Back then, all our study patients received 80% inspired oxygen as standard of care according to the recommendation of the World Health Organization to reduce the risk of surgical side infections (Allegranzi et al. 2016). However, there is no convincing evidence that supplemental inspiratory oxygen promotes formation of clinically significant atelectasis (Akca et al. 1999) or worsens postoperative oxygenation (Cohen et al. 2019). More advanced methods to assess lung injury, as the measurement of exhaled biomarkers or extravascular lung water, could have possibly provided more accurate information about the actual impact of our given fluid. We have chosen this method as it is noninvasive, feasible, can easily be implemented in daily clinical practice, and has already been used in several clinical trials (Treschan et al. 2017; Treschan et al. 2012; Holte et al. 2007a; Holte et al. 2003; Zoremba et al. 2010; Larson et al. 2009). A recent study showed an association of postoperative pulmonary parameters with postoperative pulmonary complications (Treschan et al. 2017). Also, our spirometry results are consistent with the findings of our main trial, in which goal-directed colloid administration did not affect a composite of postoperative complications and in particular the risk of pulmonary complications compared to crystalloid administration (Kabon et al. 2019). Despite the fact that the incidence of pulmonary complications did not show any significant difference between the groups, the results of this preplanned substudy might serve to further emphasize the findings of our main trial.

Within our goal-directed fluid regimes, we compared a balanced to an unbalanced solution. We have chosen Voluven bolus administration as this reflected standard of care for goal-directed fluid therapy at our institution. While the infusion of larger doses of unbalanced solutions might lead to hyperchloremic acidosis, our administered amount of unbalanced colloid infusion resulted in similar base excess values and low-grade acidosis compared to the balanced crystalloid group. Another potential limitation is that we did not control fluid management during the postoperative study period. However, as fluid intake during the first two postoperative days was comparable between the two groups, it is unlikely that postoperative fluid management might have affected our outcomes. We also measured arterial oxygen tension and arterial oxygen saturation only 6 h after surgery, but not during the further postoperative course. Thus, we are not able to provide any longitudinal data about the potential effect of our fluid management on alveolar-arterial oxygen diffusion.

A further limitation is the small sample size, and that our study might be underpowered due to the fact that the fluid-sparing effect of colloids and thus the ratio between the two infused solutions has been lower than previously expected. Moreover, a considerable number of patients was not able to perform spirometry tasks at all measurement points.

Lastly, there was a long enrollment period in our main trial and a consequently prolonged time interval between recruitment of the last patient in this substudy and submission of our current results, which was only feasible after publication of the main trial (Kabon et al. 2019). To the extent that practice changes, results might be less relevant to present patients. Specifically, we selected hydroxyethyl starch 130/0.4 as this was the most commonly used colloid within goal-directed treatment algorithms, when we designed our main trial. However, due to concerns of possible renal toxicity in critically ill patients, which have been raised by international regulatory authorities in 2013, clinicians widely preferred alternative colloids for volume replacement therapy. Still, goal-directed fluid management represents current standard of care, and HES remained approved for fluid resuscitation in case of hypovolemia in perioperative non-septic patients. Thus, our results can still be extrapolated to current clinical practice. Nevertheless, results might differ with alternative colloids.

## Conclusion

Despite a fluid-sparing effect, goal-directed administration of hydroxyethyl starch showed no beneficial effect on postoperative pulmonary function parameters compared to a goal-directed crystalloid administration. This suggests that, at least in regard to pulmonary function, it might not matter which type of fluid is administered as long as it is tailored to the individual needs of patients.

## Data Availability

barbara.kabon@meduniwien.ac.at

## Abbreviations

ASD: Absolute standardized difference
BMI: Body mass index
FEV1: Forced expiratory volume in 1 second
FiO_2_: Fraction of inspired oxygen concentration
FTc: Corrected flow time
FVC: Forced vital capacity
ICU: Intensive care unit
MAP: Mean arterial blood pressure
PaCO_2_: Arterial partial pressures of carbon dioxide
PACU: Postoperative care unit
PCA: Patient-controlled analgesia
PaO_2_: Arterial partial pressures of oxygen
PEEP: Positive end-expiratory pressure
PEF: Peak expiratory flow
SV: Stroke volume
TWA: Time-weighted average
